# Sex difference in clot lysability and association to coronary artery calcification

**DOI:** 10.1186/s13293-018-0168-8

**Published:** 2018-02-13

**Authors:** Ramshanker Ramanathan, Niels Peter R. Sand, Johannes J. Sidelmann, Bjarne L. Nørgaard, Jørgen B. Gram

**Affiliations:** 1 Department of Cardiology, Hospital of South West Denmark, Esbjerg, Denmark; 2 Department of Regional Health Research, University of Southern Denmark, Esbjerg, Denmark; 3 Department of Clinical Biochemistry, Hospital of South West Denmark, Esbjerg, Denmark; 4 Unit for Thrombosis Research, Department of Regional Health Research, University of Southern Denmark, Esbjerg, Denmark; 5 Department of Cardiology, Skejby University Hospital, Aarhus, Denmark

**Keywords:** Sex difference, Cardiovascular disease, Coronary artery calcification, Fibrin clot lysability

## Abstract

**Background:**

Incidence and prevalence of cardiovascular disease (CVD) differ between sexes, and women experience CVD later than men. Changes in fibrin clot lysability are associated with CVD, and the present study addresses sex differences in fibrin clot lysability in asymptomatic middle-aged individuals and the relation to coronary artery calcification (CAC).

**Methods:**

Participants free of morbidities and medication, *N* = 163, were randomly chosen from a national registry among citizens, 50 or 60 years of age, and were followed for 5 years. CAC was determined by the Agatston (Ag) score both at baseline and at follow-up. Based on the changes in Ag, the population was divided into two groups: ΔAg = 0 U or ΔAg > 0 U. Fibrin clot analyses were based on turbidimetric methods.

**Results:**

At baseline, 116 women and 97 men were included; 84 women and 79 men completed the 5-year follow-up (77%). Independently of covariates, women with ΔAg > 0 had reduced mean (SD) fibrin lysability at follow-up, 40.2% (15.9), both in comparison to baseline, 47.8% (20.4), *p* = 0.001, to women with ΔAg = 0 U, 51.2% (24.5), *p* = 0.028, and to men with ΔAg > 0 U, 54.4% (21.0), *p* = 0.002.

**Conclusions:**

Fibrin clot lysability changes over time with considerable sex differences. Women with progression of CAC have reduced fibrin clot lysability compared to men, indicating a sex-specific association between morphological vessel wall changes and fibrin clot lysability.

## Background

The incidence and prevalence of cardiovascular disease (CVD) differ between sexes, and women experience CVD later than men [1]. These epidemiological observations have been the fundament for studies searching for sex differences in biochemical [2] and morphological [3] characteristics in the subclinical phase of the disease.

The preponderance of studies have shown that premenopausal women have a favorable lipid profile, lower blood pressure levels, and less coronary artery calcification (CAC) compared with age-matched men [4, 5]. After the menopause the condition changes towards alignment with the age-dependent changes observed in men [6], probably due to the lack of the protecting effects of estrogens in post-menopausal women [7, 8]. Studies have indicated that although the debut of CVD is postponed, the short-term mortality after myocardial infarction (MI) is higher in women younger than 75 years old than in age-matched men [9]. The causes of these findings are unclear [10], but fibrin may in this context play an important role, as it is a matrix for tissue repair and has been shown to contribute to plaque growth in a multitude of ways [11–13]. Therefore, it might be of relevance to explore alterations in fibrin clot lysability in relation to development of CAC as a surrogate marker of CVD.

More than two decades ago, Fatah et al. observed a connection between abnormal fibrin architecture in men with MI compared to healthy controls [14]. Since then, studies have elaborated the link between CVD and altered fibrin structure and clot lysability in different patient categories [15–19]. Most of these studies have shown that defect fibrin clot lysability is associated with the evolution of CVD [15, 17, 20, 21]. However, sex differences in fibrin clot lysability have not been thoroughly evaluated, and the relation to coronary atherosclerosis is still speculative.

Consequently, in the present cross-sectional cohort study, we examined sex differences and changes over time in fibrin clot lysability in asymptomatic middle-aged individuals. Furthermore, we addressed the potential association between fibrin clot lysability and CAC.

## Methods

### Study design and participants

This cross-sectional cohort study with follow-up recruiting participants from the Danish Risk Score (DanRisk) study [22] was performed at four hospitals (Svendborg, Odense, Vejle, and Esbjerg) in Southern Denmark from 2009 to 2010. Study participants were randomly chosen from the Danish Central Person Registry among citizens born either in 1949 or 1959. The present study focuses on participants recruited from Esbjerg [23]. In brief, 458 participants were invited at baseline, of whom 329 (72%) accepted the invitation. After exclusion of individuals with symptomatic CVD (angina, myocardial infarction, treatment with percutaneous coronary revascularization or coronary by-pass surgery), atrial fibrillation, stroke, peripheral artery disease, heart valve disease, and diabetes or taking any kind of medication, a total of 213 (47%) comprised the study population at baseline. After 5 years, all 213 participants were re-invited to a follow-up assessment, and 163 (77%) participants were re-examined and thus constituted the final population.

Prior to examination, the participants filled in a questionnaire concerning medical history, family history with CVD, and smoking habits. In addition, participants were interviewed about the same topics. Physical examination included BMI calculations based on measurements of height and weight, waist circumference, blood pressure and pulse (average of three measurements after 5 min of rest), and an ECG.

### Cardiac CT

All CT scans both at baseline and at follow-up were analyzed by the same experienced cardiologist, who was blinded to all other patient data. CAC was expressed as Agatston (Ag) score [24]. The internal validity of the calcium scoring was high [22]. Non-contrast scan data were acquired during an inspiratory breath hold.

Ag at baseline was assessed by a Toshiba 64-slice CT-scanner (Aquilion, Toshiba Medical Systems). The following technical settings were used: gantry rotation time 450 ms, collimation 64 × 0.5 mm, slice thickness 3 mm, 120 kV tube voltage, 240 mA tube current, and prospective gating at 75% of the R-R interval. At follow-up, Ag was assessed using a Philips 64-slice scanner (Brilliance 64, Philips Healthcare). The following technical settings were used: gantry rotation time 400 ms, collimation 64 × 0.625 mm, slice thickness 2.5 mm, 120 kV tube voltage, 220 mA tube current, and prospective gating at 75% of the R-R interval. The mean (SD, range, *k*-factor) estimated radiation dose at baseline was 1.36 mSv (0.54, 0.49–3.06 mSv, 0.0145), and the mean (SD, range, *k*-factor) estimated radiation dose at follow-up was 1.09 mSv (0.31, 0.48–2.22 mSv, 0.0145).

The study population was separated into two groups based on the numeric difference between Ag at follow-up and baseline: ΔAg = 0 U or ΔAg > 0 U.

### Blood collection and handling

Blood samples were drawn from an antecubital vein into sterile vacuum plastic tubes containing either 0.109 mol/L citrate or no anticoagulants, and the plasma or serum isolated after 20 min of centrifugation at 2000*g* at 20 °C were frozen and stored in aliquots at − 80 °C until analysis.

### Biochemical analysis

Lipids were analyzed using Cholesterol, Direct LDL, Ultra HDL, and Triglycerides kits employing the Architect C16000 analyzer. Kits and analyzer were from Abbott, Wiesbaden, Germany. Concentrations of CRP and fibrinogen were determined on a BN-II nephelometer using antibodies and reagents from Siemens Healthcare Diagnostics GmbH, Marburg, Germany. Plasminogen activator inhibitor type 1 (PAI-1) antigen, was quantitatively determined by a sandwich enzyme immunoassay (ELISA) using in-house specific antibodies as previously described [25] employing a microplate reader (Tecan Trading AG, Basle, Switzerland).

Fibrin clot lysis was recorded using turbidity measurements as previously described [20]. In brief, citrate-stabilized plasma was mixed with thrombin, CaCl_2_, and Tween 80 and incubated overnight in a microtiter plate sealed with adhesive tape. All clots were made in duplicate. To initiate fibrinolysis, t-PA and flufenamic acid were added onto the clots. The 405-nm optical density (OD) was then followed on a Sunrise plate reader (Tecan Austria, Grödig/Salzburg, Austria) every 5 min for 4 h at 25 °C. The rate of fibrinolysis per hour was determined from the slope of the curve at the time when the slope became constant and was normalized with respect to the maximum absorbency value before lysis initiation.

### Statistics

Continuous variables are presented as means and standard deviations (SD) or median and interquartile ranges (IQR) as appropriate. Normality was examined by the Shapiro-Wilk test as well as by visually assessing histograms and Q-Q plots. If possible, non-normally distributed variables were log-transformed to achieve normality. Dichotomous variables are shown as numbers and percentages. Student’s *t* test was used for comparison of normally distributed continuous variables and the Mann-Whitney test for comparison of non-normally distributed continuous variables. The *χ*^2^ test was performed for comparison of dichotomous variables. The paired Student’s *t* test was used for paired comparison of normally distributed continuous variables, and the Wilcoxon signed rank test was used for paired comparison of non-normally distributed continuous variables. *p* values < 0.05 were considered statistically significant. Covariates were identified as variables which were significantly different between the sexes in the univariate analyses and correlated with fibrin clot lysability using the Spearman correlation test. A linear regression model using fibrin clot lysability as the dependent variable was created, and the model was adjusted for identified covariates. To ensure model validation, Q-Q plots for residuals were inspected for normality. Stata 15.0, StataCorp, TX, USA, was used for statistical analyses.

## Results

### Baseline and follow-up characteristics

Participants were followed for a mean (range) of 5.5 years (5.2–5.7 years). At baseline, 116 women and 97 men were included and 84 women and 79 men completed the follow-up. Study population characteristics at baseline and follow-up are shown in Tables 1 and 2. Women had lower levels of systolic and diastolic blood pressures, lower levels of triglycerides, higher high-density lipoprotein (HDL) cholesterol levels, and lower Ag compared to men (all *p* <  0.05) both at baseline and follow-up, while C-reactive protein (CRP) was comparable between men and women. Body mass index (BMI) was lower in women at baseline (*p* = 0.039) but comparable among men and women at follow-up, while fibrinogen was higher in women at baseline (*p* = 0.008) and comparable among men and women at follow-up. ΔAg was significantly lower in women than in men (*p* = 0.012). ΔAg = 0 U was observed in 85 participants (53 women). ΔAg > 0 U was observed in 78 participants (31 women); of these, 23 participants (10 women) had ΔAg = 1–10 U, 35 participants (12 women) had ΔAg = 11–100, and 20 participants (9 women) had ΔAg > 100 U. Negative ΔAg observed in eight participants was classified as ΔAg = 0 U.

**Table 1:** Study population characteristics at baseline

Baseline, *N* = 163	Men (*n* = 79)	Women (*n* = 84)	*p* value
Born			
1949 (*n* = 70)	30 (43)	40 (57)	
1959 (*n* = 93)	49 (53)	44 (47)	0.21
Tobacco use			
Never (*n* =77)	35 (45)	42 (55)	
Prior/current (*n* = 86)	44 (51)	42 (49)	0.53
Systolic blood pressure, mmHg Diastolic blood pressure, mmHg	140 (15) 82 (9)	132 (19) 78 (9)	0.007 0.002
Body mass index, kg/m^2^	27.4 (3.6)	26.1 (4.6)	0.039
Agatston score, U	0 (0–33)	0 (0–7)	0.012
Total cholesterol, mmol/l LDL cholesterol, mmol/l HDL cholesterol, mmol/l Triglycerides, mmol/l	5.42 (0.97) 3.34 (0.82) 1.26 (1.02–1.54) 1.47 (0.80)	5.54 (1.07) 3.23 (0.91) 1.52 (1.35–1.85) 1.14 (0.60)	0.44 0.39 < 0.001 0.003
C-reactive protein, mg/l	0.74 (0.36–1.61)	0.72 (0.29–1.86)	0.67
Fibrinogen, μmol/l	8.9 (7.9–9.9)	9.6 (8.5–10.9)	0.008
PAI-1, ng/ml	16.5 (11.2–24.4)	12.5 (6.7–20.2)	0.016
Fibrin clot lysability, %	54.8 (19.7)	51.5 (22.2)	0.32

Values are presented as mean (SD), median (IQR), or number (%). Information on C-reactive protein is missing on three participants. Information on PAI-1 is missing on four participants

*LDL* low-density lipoprotein, *HDL* high-density lipoprotein, PAI-1 plasminogen activator inhibitor type 1

**Table 2:** Study population characteristics at follow-up

Follow-up, *N* = 163	Men (*n* = 79)	Women (*n* = 84)	*p* value
Born			
1949 (*n* = 70)	30 (43)	40 (57)	
1959 (*n* = 93)	49 (53)	44 (47)	0.21
Tobacco use			
Never (*n* = 76)	34 (45)	42 (55)	
Prior/current (*n* = 87)	45 (52)	42 (48)	0.47
Systolic blood pressure, mmHg Diastolic blood pressure, mmHg	140 (17) 81 (8)	133 (16) 72 (9)	0.013 < 0.001
Body mass index, kg/m^2^	28.1 (4.2)	26.9 (4.7)	0.063
Agatston score, U	9 (0–115)	0 (0–26)	0.002
ΔAgatston score, U	4 (0–49)	0 (0–10)	0.012
Total cholesterol, mmol/l LDL cholesterol, mmol/l HDL cholesterol, mmol/l Triglycerides, mmol/l	5.31 (0.85) 3.33 (0.80) 1.30 (1.10–1.40) 1.82 (0.97)	5.54 (0.92) 3.30 (0.93) 1.55 (1.40–1.75) 1.50 (0.89)	0.10 0.80 < 0.001 0.032
C-reactive protein, mg/l	0.74 (0.46–1.61)	0.70 (0.30–2.18)	0.74
Fibrinogen, μmol/l	8.9 (7.7–10.3)	9.5 (8.4–11.1)	0.056
PAI-1, ng/ml	21.7 (15.4–27.5)	20.1 (15.0–30.3)	0.69
Fibrin clot lysability, %	52.1 (20.5)	47.2 (22.3)	0.14

Values are presented as mean (SD), median (IQR), or number (%). Information on Agatston score is missing on one participant

*LDL* low-density lipoprotein, *HDL* high-density lipoprotein, *PAI-1* plasminogen activator inhibitor type 1

### Fibrin clot lysability

**Fig. 1:**
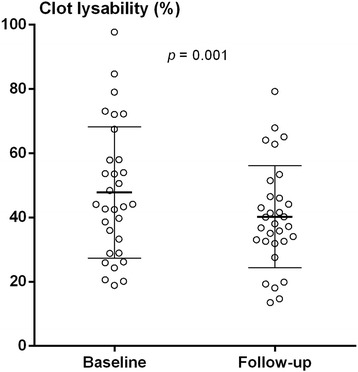
Clot lysability in women with ΔAgatston score > 0 U at baseline and follow-up (*N* = 31). Variables are compared using the paired Student’s *t* test. Mean and SD are indicated in bars

**Fig. 2:**
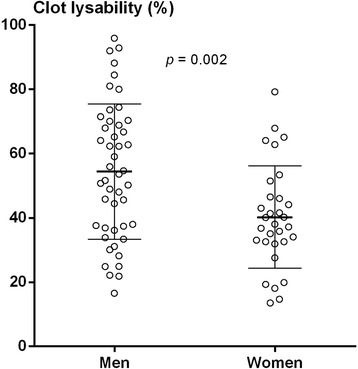
Clot lysability in men (*N* = 47) and women (*N* = 31) with ΔAgatston score > 0 U at follow-up. Variables are compared using the unpaired Student’s *t* test. Mean and SD are indicated in bars

**Fig. 3:**
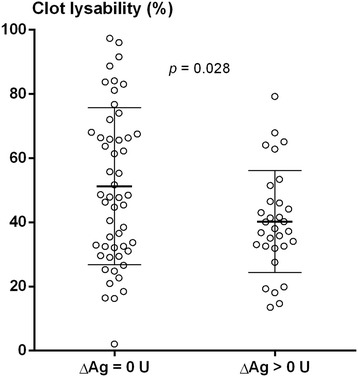
Clot lysability in women with ΔAgatston score = 0 U (*N* = 53) and ΔAgatston score > 0 U (*N* = 31) at follow-up. Variables are compared using the unpaired Student’s *t* test. Mean and SD are indicated in bars

Women with ΔAg > 0 U had reduced mean (SD) clot lysability at follow-up compared to baseline, 40.2% (15.9) versus 47.8% (20.4) (*p* = 0.001) (Fig. 1). There was no difference in clot lysability between baseline and follow-up in women with ΔAg > 0 U or in men. As shown in Figs. 2 and 3, at follow-up, women with ΔAg > 0 U had reduced mean (SD) clot lysability, 40.2% (15.9), compared to men with ΔAg > 0 U, 54.4% (21.0) (*p* = 0.002), and compared to women with ΔAg = 0 U, 51.2% (24.5) (*p* = 0.028). No difference in fibrin clot lysability between men with ΔAg = 0 U and ΔAg > 0 U was observed. Only triglycerides (*r* = − 0.168, *p* = 0.032), fibrinogen (*r* = − 0.444, *p* <  0.001), and PAI-1 (*r* = − 0.223, *p* = 0.004) correlated with fibrin clot lysability. Adjusting for the confounding effect of fibrinogen, triglycerides, and PAI-1 showed that the observed reduced fibrin clot lysability in women with ΔAg > 0 U compared to women with ΔAg = 0 U and men with ΔAg > 0 U was independent of covariation (*p* = 0.028 and *p* = 0.013, respectively).

## Discussion

In the present prospective cross-sectional cohort study, comprising apparently healthy middle-aged individuals, we demonstrate significantly reduced fibrin clot lysability during a 5-year follow-up in women with progression of CAC. Furthermore, women with progression of CAC had reduced clot lysability compared to men as well as compared to women without progression of CAC, as these subgroups had no significant alterations in clot lysability over time. These findings were independent of confounding covariates.

Although several hypotheses have been suggested as the potential cause for sex differences in the natural history of CVD, basic mechanisms are still unknown. In this perspective, a positive correlation between high fibrinogen levels in young adulthood and incidence of CAC later in life has been reported in the Coronary Artery Risk Development in Young Adults (CARDIA) study [13], and other studies have reported an association between high fibrinogen levels and thrombosis [26]. In general, women have higher plasma concentration of fibrinogen than age-matched men [27], and fibrinogen as a precursor of fibrin is considered to be an important determinant of the fibrin structure [28]. A prothrombotic effect of increased fibrinogen is probably closely linked to alterations in fibrin structure [29, 30] causing fibrin to be more resistant to lysis. In the present study, we observed differences in fibrinogen concentrations between sexes at baseline but not at follow-up, and although the fibrinogen concentration correlated with clot lysability, the sex-related differences in clot lysability remained significant after adjustment for the potential confounding effect of fibrinogen. These findings indicate that other factors than fibrinogen are responsible for the observed differences in fibrin clot lysability.

We have previously shown that women with atrial fibrillation differ in fibrin polymerization characteristics compared to men [18] and that women receiving OC have alterations in fibrin structure and increased clot lysability [31]. Moreover, middle-aged women receiving estrogen therapy have a reduced burden of CAC compared to women receiving placebo [32]. We therefore hypothesize that the reduced wall repair mechanisms and increasing CAC observed in women after menopause [8] may partly be due to altered fibrin lysability. Thus, a reduction of more than 20% in fibrin clot lysability recorded in women with CAC progression may contribute to the different cardiovascular pathophysiology observed in women after the menopause.

In addition, clinical studies suggest that fibrinogen and fibrin deposits in atherosclerotic plaques, and not circulating fibrinogen, contribute to plaque growth and stability by stimulating migration and proliferation of smooth muscle cells as well as creating a matrix for the calcium deposits in the growing plaque [11–13]. As both fibrinogen and fibrin are present in early as well as advanced atherosclerotic plaques [33], a reduced fibrin clot lysability may play a central role in plaque growth and remodeling, with important influence in wound healing and vascular remodeling [34]. In the modern context of Virchow’s triad [35], the present study might therefore be of interest, as we report a correlation between CAC as an abnormality in the vessel wall and a concomitant reduced clot lysability occurring only in women.

### Limitations

Negative ΔAg was recorded in eight participants and was registered as ΔAg = 0 U, and of these, five participants had a decrease in Ag ≥ 10 U. However, repeat analysis after exclusion of all eight participants did not change our findings. Several studies report difficulties in assessing CAC progression and definition of subgroups based on progression in CAC [36–39]. Our subgroup analysis showed a trend towards decreased fibrin clot lysability with increasing ΔAg in women compared to men; however, the number of participants in each group is limited. Further research in a larger population is needed to perform subgroup analyses. The hormonal status in the women was unknown, but no significant differences in clot lysability between the two age cohorts in women at both baseline and follow-up were observed. At the study inclusion, women taking any kind of hormone replacement therapy were excluded. Scanners from different vendors were used to assess CAC at baseline and follow-up. However, according to Willemink et al., both the median relative difference of Ag and the reclassification rate between the Philips and Toshiba scanners used in the present study were low [40]. Fifty study participants were not re-examined at follow-up, and clinical information on these participants are not available. However, these participants were comparable to the study participants who were re-assessed in terms of cardiovascular risk factors and Ag at baseline.

## Conclusion

Women with progression of CAC over 5 years have reduced clot lysability compared to men. In addition, these women have reduced clot lysability compared to women with no development of CAC. This study suggests that fibrin clot lysability plays a role in the evolution of CVD.

## Abbreviations

Ag: Agatston score
BMI: Body mass index
CAC: Coronary artery calcification
CAD: Coronary artery disease
CARDIA: Coronary Artery Risk Development in Young Adults study
CRP: C-reactive protein
CT: Computed tomography
CVD: Cardiovascular disease
HDL: High-density lipoprotein
IQR: Interquartile range
LDL: Low-density lipoprotein
MI: Myocardial infarction
OC: Oral contraceptives
SD: Standard deviation
